# Transmission-Blocking Vaccines: Focus on Anti-Vector Vaccines against Tick-Borne Diseases

**DOI:** 10.1007/s00005-014-0324-8

**Published:** 2014-12-12

**Authors:** Girish Neelakanta, Hameeda Sultana

**Affiliations:** Center for Molecular Medicine, Department of Biological Sciences, Old Dominion University, Norfolk, VA 23529 USA

**Keywords:** Transmission-blocking vaccine, Anti-vector vaccine, *Ixodes*, *Dermacentor*, *Amblyomma*, *Haemaphysalis*, *Rhipicephalus*, *Ornithodoros* species

## Abstract

Tick-borne diseases are a potential threat that account for significant morbidity and mortality in human population worldwide. Vaccines are not available to treat several of the tick-borne diseases. With the emergence and resurgence of several tick-borne diseases, emphasis on the development of transmission-blocking vaccines remains increasing. In this review, we provide a snap shot on some of the potential candidates for the development of anti-vector vaccines (a form of transmission-blocking vaccines) against wide range of hard and soft ticks that include *Ixodes*, *Haemaphysalis*, *Dermacentor*, *Amblyomma*, *Rhipicephalus* and *Ornithodoros* species.

## Introduction

Arthropod-borne diseases account for severe morbidity and mortality in worldwide human population (Githeko et al. 2000; Hill et al. 2005). Based on the disability-adjusted life year (DALY) estimates, infections associated with one-sixth of the human population are due to arthropod-borne diseases (Hill et al. 2005; WHO 2004). World Health Organization (WHO) reports malaria to be the leading vector-borne disease in the world followed by Leishmaniasis, Trypanosomiasis, Yellow fever, Dengue, Chagas disease and Japanese Encephalitis (Hill et al. 2005; WHO 2004, 2013, 2014). The recent epidemics of historically recognized diseases such as Tick-borne encephalitis, Kyasanur forest disease, Crimean-Congo hemorrhagic fever and Rocky mountain spotted fever suggests increase in the scope and magnitude of tick-borne diseases world wide (Demma et al. 2006; Maltezou et al. 2010; Pattnaik 2006; Randolph 2008). Even though tick-borne diseases are considered pale in comparison to the other arthropod-borne diseases, the steady increase in the annual incidence of some of the tick-borne diseases such as Lyme disease, human anaplasmosis and human monocytic ehrlichiosis implies a potential threat to human health (CDC 2014). Some of the important tick-borne diseases that occur worldwide are listed in Table 1. No vaccines or effective therapies are available to treat several of these important vector-borne diseases.

**Table 1:** Worldwide tick-borne diseases

Tick-borne diseases	Agent	Tick species	Tick family
Lyme Borreliosis	*Borrelia burgdorferi*	*Ixodes scapularis*	Ixodidae
		*I. dentatus*	Ixodidae
		*I. pacificus*	Ixodidae
	*B. afzelii*	*I. ricinus*	Ixodidae
		*I. persulcatus*	Ixodidae
		*I. nipponensis*	Ixodidae
	*B. garinii*	*I. ricinus*	Ixodidae
		*I. uriae*	Ixodidae
		*I. hexagonus*	Ixodidae
		*I. trianguliceps*	Ixodidae
		*I. persulcatus*	Ixodidae
		*Haemaphysalis longicornis*	Ixodidae
Human Anaplasmosis	*Anaplasma phagocytophilum*	*I. scapularis*	Ixodidae
		*I. pacificus*	Ixodidae
Human Babesiosis	*Babesia microti*	*I. scapularis*	Ixodidae
Tularemia	*Francisella tularensis*	*I. dentatus*	Ixodidae
		*I. ricinus*	Ixodidae
		*Dermacentor variabilis*	Ixodidae
		*D. andersoni*	Ixodidae
		*Amblyomma americanum*	Ixodidae
Q-fever	*Coxiella burnetii*	*I. dentatus*	Ixodidae
		*I. trianguliceps*	Ixodidae
		*Rhipicephalus sanguineus*	Ixodidae
		*A. americanum*	Ixodidae
Japanese spotted fever	*Rickettsia japonica*	*I. ovatus*	Ixodidae
		*D. taiwanensis*	Ixodidae
		*H. flava*	Ixodidae
Queensland tick typhus	*R. australis*	*I. holocyclus*	Ixodidae
Tick-borne encephalitis	Tick borne encephalitis virus	*I. ricinus*	Ixodidae
		*I. persulcatus*	Ixodidae
Boutonneuse fever	*R. conorii*	*R. sanguineus*	Ixodidae
Ehrlichiosis	*Ehrlichia chaffeensis*	*A. americanum*	Ixodidae
	*E. ewingii*	*A. americanum*	Ixodidae
Rocky mountain spotted fever	*R. rickettsii*	*A. canjennense*	Ixodidae
		*D. variabilis*	Ixodidae
		*D. andersoni*	Ixodidae
		*R. sanguineus*	Ixodidae
African tick-bite fever	*R. africae*	*A. hebraeum*	Ixodidae
Siberian tick typhus	*R. sibirica*	*D. nuttalli*	Ixodidae
Relapsing fever	*B. duttoni*	*Ornithodoros moubata*	Argasidae
	*B. hermsii*	*O. hermsi*	Argasidae
	*B. turicatae*	*O. turicata*	Argasidae
African relapsing fever	*B. crocidurae*	*O. erraticus sonrai*	Argasidae

There are about 5–19 million species of arthropods existing in the world of which some serve as vectors for various pathogens that cause diseases in humans (Ødegaard 2000). Arthropods have become most successful to serve as competent vectors for disease transmission due to their capacity of biting host, ingesting blood meal from hosts and permitting pathogen survival in them for a longer period of time (Desenclos 2011; Goddard 2008). Several studies have reported use of live-attenuated parasite vaccine candidates to control arthropod-borne disease pathogenesis in various animal models (Barry et al. 2009; Callow 1978; Conlan 2011; Gardner and Ryman 2010; Heinz and Stiasny 2012; Orlinger et al. 2011; Reed et al. 2014; Sultana et al. 2009; Wang et al. 2014a; Yun and Lee 2014). However, limited of them are successful and approved for human use (Conlan 2011; Gardner and Ryman 2010; Orlinger et al. 2011; Yun and Lee 2014). Therefore, effective strategies need to be developed in order to combat both arthropods and pathogens. Of many strategies (Coutinho-Abreu et al. 2009; Oliveira et al. 2009; Thomas and Read 2007; Valenzuela 2004a, b), development of transmission-blocking vaccines has provided a significant leap that has moved research in this field forward for clinical trials (Malkin et al. 2005; Saul et al. 2007; Wu et al. 2008).

Anti-vector vaccines are a type of transmission-blocking vaccines aimed to target vector molecules to block pathogen transmission from arthropods to mammalian hosts (Billingsley et al. 2008; Coutinho-Abreu and Ramalho-Ortigao 2010; Coutinho-Abreu et al. 2009; Oliveira et al. 2009; Valenzuela 2004a). Several features are important to be considered for the development of anti-vector vaccines for humans (de la Fuente and Merino 2013; Merino et al. 2013). First, candidate molecule should be critical for vector–pathogen interaction. Disruption of the candidate molecule by gene knock-out/down, RNA interference or antibody blocking should affect acquisition or transmission or replication of pathogen inside vector. Second, the primary amino acid sequence of the candidate molecule should be highly conserved among different isolates of that species to facilitate development of unique antigen for vaccine design. Third, candidate molecule should provide high antibody titer upon injection into humans to block pathogen transmission from vectors. Fourth, candidate molecule should be compatible with different adjuvants to effectively induce immune responses in humans. Lastly, candidate molecule should not result in exaggerated immune responses leading to immune-related disorders in humans.

**Fig. 1:**
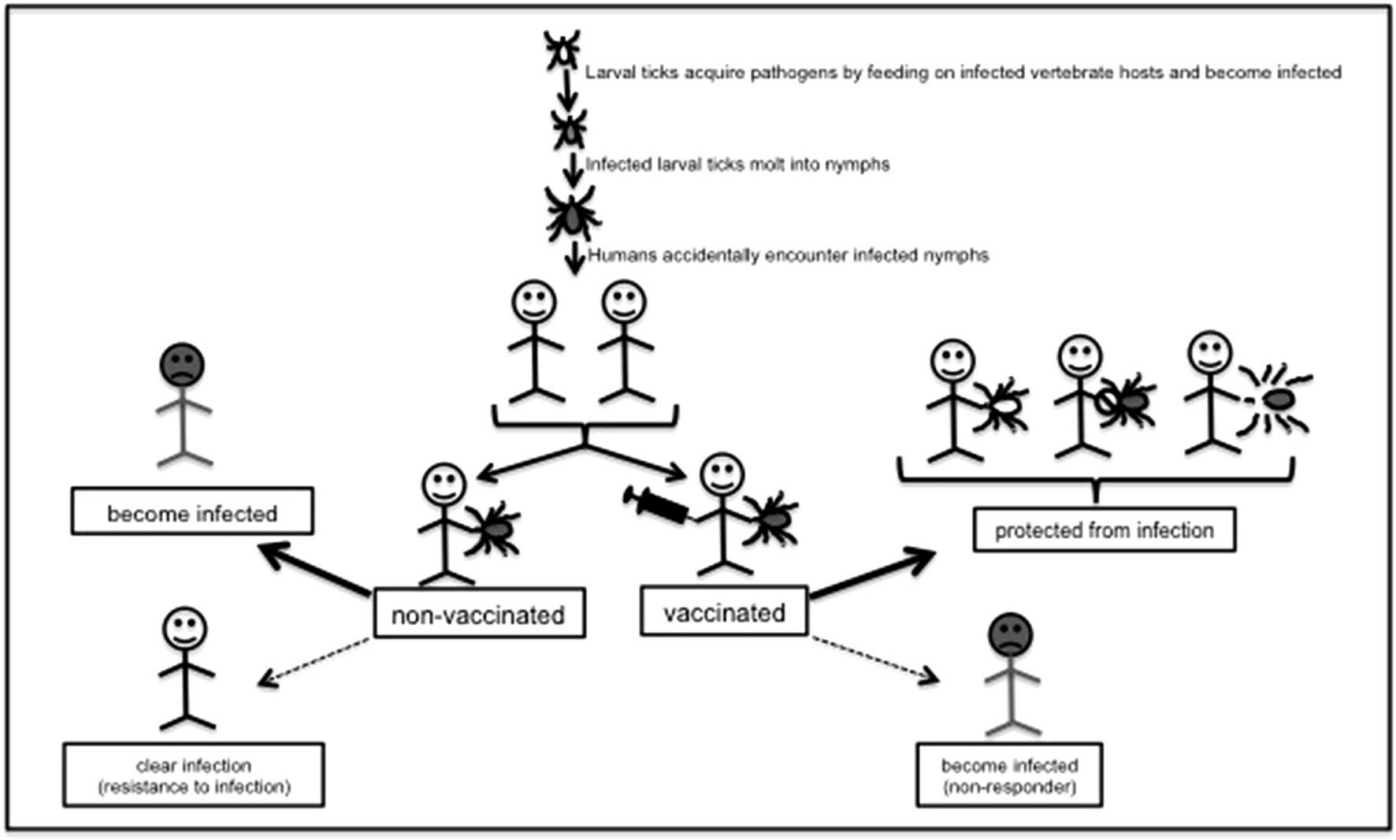
Anti-vector vaccines. Schematic representation of the proposed strategy on the effect of anti-tick vaccination in humans is shown. Larval ticks become infected by feeding on infected vertebrate hosts and molt into nymphs. Nymphs transmit pathogens to humans. Several scenarios might be envisioned with regard to the effect of anti-tick vaccine administration in natural human population. Non-vaccinated humans bitten by infected ticks may become infected and develop disease symptoms or remain uninfected by natural resistance. On the other hand, vaccinated individuals might be protected from infection or may still become infected due to poor responsiveness to the vaccine. As shown in the illustration, three types of vaccine strategies can be considered. First, anti-vector vaccine can be designed to degrade pathogens inside ticks. Second, anti-vector vaccine can be designed to target vector molecules to prevent pathogen transmission. Third, anti-vector vaccine can be designed to target vector molecules to degrade or affect physiology and survival of ticks

The basic proposed strategy on the effect of anti-vector vaccination in humans is illustrated in Fig. 1. For instance, in the Northeastern part of the United States, *Ixodes scapularis* ticks transmit *Borrelia burgdorferi* the causative agent of Lyme disease, *Anaplasma phagocytophilum* the agent of human anaplasmosis and *Babesia microti* the agent of human Babesiosis (Table 1) (Anderson and Magnarelli 2008). In nature, *I. scapularis* larval ticks get infected with these pathogens upon feeding on infected vertebrate hosts (Sonenshine and Roe 2014). Infected larval ticks molt into nymphs. Humans accidently become infected upon bite by an infected nymph carrying these pathogens (Fig. 1). Three types of anti-vector vaccine development strategies can be proposed to protect humans from being infected. First, anti-vector vaccine can be designed to degrade pathogens inside ticks that subsequently make ticks free of pathogens (Fig. 1). Second, anti-vector vaccine can be developed against one of the vector molecule to prevent transmission of pathogens to humans (Fig. 1). Third, anti-vector vaccines can be designed to degrade or affect physiology of ticks upon ingestion of a blood meal. Collectively, these proposed strategies would significantly improve development of potential therapeutic targets to treat and/or control several of the human vector-borne diseases.

In this review, we focus on some of the important findings in the field of anti-vector vaccines against wide range of hard and soft ticks that include *Ixodes*, *Haemaphysalis*, *Dermacentor*, *Amblyomma*, *Rhipicephalus* and *Ornithodoros* species. Due to our focus on providing a “snap shot” with certain level of subjectivity of including studies from both hard and soft ticks, several of the other important findings in this field are not included.

### Anti-*Ixodes* Vaccine Candidates

Several of the studies have reported immunization of animals with tick antigens to study the effect of vaccination on pathogen transmission and acquisition. Active immunization of mice with Salp15, a 15-kDa secreted salivary gland protein from *I. scapularis* showed substantial protection (60 %) from tick-borne *Borrelia* (Dai et al. 2009). Salp15 protein has several immunosuppressive properties (Juncadella and Anguita 2009). It inhibits CD4^+^ T cell activation, complement activity and dendritic cell activation (Juncadella and Anguita 2009). *B. burgdorferi* coats itself with Salp15 protein during exit from ticks (Ramamoorthi et al. 2005). This coating prevents evasion of immune responses against spirochetes upon entry into the mammalian host (Ramamoorthi et al. 2005). Interaction of Salp15 with *B. burgdorferi* OspC is critical during this process (Ramamoorthi et al. 2005). Immunization of mice with Salp25D, a 25-kDa salivary gland protein from *I. scapularis* reduced spirochete acquisition by ticks to threefold in comparison to the non-immunized controls (Narasimhan et al. 2007). Salp25D has significant homology to peroxiredoxins antioxidants that has been shown to play significant role in protecting *B. burgdorferi* from reactive oxygen produced by neutrophils (Narasimhan et al. 2007). Blockade of gut-specific protein TROSPA (tick receptor for outer surface protein A) by TROSPA-antisera showed significant reduction (75 %) of *B. burgdorferi* adherence to tick gut in vivo that subsequently prevented colonization and transmission of spirochete from ticks to mice (Pal et al. 2004). Interaction of TROSPA with *B. burgdorferi* Outer surface protein A (OspA) is critical for colonization of spirochetes in the gut (Pal et al. 2004). Mice immunized with tick histamine release factor (tHRF) also showed substantial reduction in spirochete transmission (Dai et al. 2010). Twenty to thirty percent of tHRF-immunized mice were fully protected from spirochete transmission (Dai et al. 2010). Recently, the tick salivary lectin pathway inhibitor (TSLPI) from *I. scapularis* was shown to prevent killing of *B. burgdorferi* from host complement system (Schuijt et al. 2011). Mice vaccinated with TSLPI showed 30 % reduction in spirochete loads, but did not completely block transmission (Schuijt et al. 2011). Due to lower percentage of protection of mice seen upon TSLPI immunization, the authors in the study proposed a combination vaccine therapy to prevent Lyme disease (Schuijt et al. 2011). In Northeastern part of the United States*, I. scapularis* ticks survive overwintering stages (Anderson and Magnarelli 2008). Recently, we identified an antifreeze glycoprotein (IAFGP) molecule in *I. scapularis* and *I. ricinus* ticks (Neelakanta et al. 2010). IAFGP is found to be critical for the survival of ticks at cold temperatures (Neelakanta et al. 2010). Interestingly, *A. phagocytophilum*-infected ticks produced more IAFGP and showed greater resistance to cold temperatures, suggesting a symbiotic relationship of this microbe with its vector host (Neelakanta et al. 2010). RNA interference of IAFGP showed significant reduction of *A. phagocytophilum* acquisition in ticks when fed on infected mice (Neelakanta et al. 2010). These results clearly elucidate the important role of IAFGP in ticks. Salp16, a 16-kDa tick salivary gland protein, is shown to be critical for *A. phagocytophilum* survival in ticks (Sukumaran et al. 2006). RNA interference (RNAi) of *salp16* expression showed significant (90 %) reduction of *A. phagocytophilum* migration from gut to salivary glands (Sukumaran et al. 2006). Our recent study showed that *A. phagocytophilum* induces tick actin phosphorylation to selectively regulate *salp16* gene expression (Sultana et al. 2010). We found substantial increase in G-actin content in tick nuclear extracts upon *A. phagocytophilum* infection that subsequently influenced *salp16* gene expression (Sultana et al. 2010). These results suggest important role of Salp16 in tick-*A. phagocytophilum* interaction (Sultana et al. 2010). Our study also demonstrated importance of *I. scapularis* p21-activated kinase, PI3-kinase and G-proteins in *A. phagocytophilum* survival in ticks (Sultana et al. 2010). Study from Labuda et al. (2006) showed that immunization of mice with 64TPR, a tick cement protein protected mice from tick-borne encephalitis virus transmission (Labuda et al. 2006). Viral titers were reduced to more than 50 % in vaccinated mice in comparison to the controls (Labuda et al. 2006). 64TPR is a 15-KDa protein that express at higher levels during tick feeding (Labuda et al. 2006). Moreover, 64TPR has been shown as a broad-spectrum vaccine candidate against several tick species (Labuda et al. 2006).

Homologues of Salp15 have been identified in *I. pacificus, I. persulcatus* and *I. sinensis* that transmit *Borrelia* (Table 1) in Western North America and Asia (Hojgaard et al. 2009; Wang et al. 2014b). Three Salp15 homologues were identified in *I. ricinus*, where one of the predicted protein showed 80 % and two other proteins showed 60 % similarity with *I. scapularis* Salp15 (Hovius et al. 2007). Recent study has identified 5 and 3 Salp15-like proteins in *I. persulcatus* and *I. sinensis* ticks, respectively (Wang et al. 2014b). Intraspecies and interspecies comparison of these proteins with other Salp15 proteins revealed equal degree of similarity (Wang et al. 2014b). High degree of similarity was evident in the 53-amino acid stretches at the C-terminus of the protein (Hojgaard et al. 2009). These studies have demonstrated that Salp15 and Salp15-like proteins are distributed in three clades and represent a multigene family (Hojgaard et al. 2009; Hovius et al. 2007; Wang et al. 2014b). Homologues of Salp16 have been identified in *I. persulcatus* Schulze ticks (Hidano et al. 2014). These proteins were designated as Salp16 Iper1 and Iper2 (Hidano et al. 2014). *I. persulcatus* Salp16 proteins have been shown to attenuate oxidative burst of the bovine neutrophils and inhibited migration induced by chemoattractant interleukin-8 (Hidano et al. 2014). This study provides important information on the role of Salp16 in tick blood feeding and as immunosuppressant of neutrophils (Hidano et al. 2014). Homologues of TROSPA have been identified in *I. ricinus* and *I. persulcatus* ticks (Konnai et al. 2012). *I. persulcatus* TROSPA was found to be 88.2 and 87.8 % identical to *I. scapularis* and *I. ricinus* TROSPA, respectively (Figlerowicz et al. 2013; Konnai et al. 2012). Figlerowicz et al. (2013) showed that *I. ricinus* recombinant TROSPA retained capacity of native protein to form a complex with OspA and induced significant level of IgG in orally immunized animals (Figlerowicz et al. 2013). Recent study has used *Anaplasma and Babesia* infection model to characterize Subolesin (SUB), SILK and TROSPA as potential antigens to control cattle tick infestations (Antunes et al. 2014). Rabbit polyclonal antibodies generated against SUB, SILK and TROSPA were added to uninfected or infected bovine blood to capillary feed *Rhipicephalus microplus* ticks (Antunes et al. 2014). Even though results from capillary feeding showed substantial effect on tick weight and oviposition, pathogen levels were unaltered in ticks treated with these antibodies in comparison to the control ticks (Antunes et al. 2014). Due to tick-to-tick variations, this study concluded that capillary feeding was not an ideal technique to characterize efficacy of a vaccine candidate (Antunes et al. 2014). Recent study analyzed whether adaptive evolution has shaped the Salp15 protein family (Schwalie and Schultz 2009). Evidence was seen at several positions within the Salp15 protein family showing phases of positive selection (Schwalie and Schultz 2009). It would be interesting to determine whether the set of several conserved proteins that function in tick–pathogen interactions is also under positive selection. Like Salp15, if Salp16, TROSPA and SUB are also under positive selection, it might be interesting to characterize whether these proteins play differential roles in the interactions with various pathogens (Table 1).

### Anti-*Haemaphysalis* Vaccine Candidates

Some of the *Haemaphysalis* species transmit pathogens to humans that cause diseases like Lyme borreliosis and spotted fever (Table 1). Study from Chai et al. (2009) has identified 12 unique cDNA sequences in a phage library that encode putative immunodominant antigens in *Haemaphysalis longicornis*. Harnnoi et al. (2006) have identified genes encoding cement-like antigens in *H. longicornis*. These cement-like proteins in *H. longicornis* were upregulated upon feeding (Harnnoi et al. 2006). HISP, a cubilin-related serine proteinase was identified in *H. longicornis* (Miyoshi et al. 2004). HISP expression was highly upregulated during tick feeding indicating its role in digestion of host blood components (Miyoshi et al. 2004). Recent study characterized acid phosphatase from *H. longicornis* (Zhang et al. 2011). Rabbits vaccinated with recombinant acid phosphatase conferred protective immunity, resulting in 28 % mortality and 10.6 % reduction in body weight of adult ticks (Zhang et al. 2011). Scavenger receptors are predominantly expressed by mammalian macrophages and are shown to be important in innate immunity (Aung et al. 2011). Study from Aung et al. (2011) have identified Scavenger receptor homologue (HISRB) in *H. longicornis* for the first time in Chelicerata that include ticks, horseshoe crabs, scorpions, spiders and mites. Silencing of HISRB gene resulted in reduced tick body weight (Aung et al. 2011). It is not surprising to hypothesize that HISRB can be considered as an effective candidate for the development of vaccine against *H. longicornis* (Aung et al. 2011). Follistatin-related protein (Zhou et al. 2006a) and Valosin-containing protein (Boldbaatar et al. 2007) have been identified in *H. longicornis* that are critical for tick oviposition and engorgement. A salivary gland protein Longistatin, a serine protease with two EF-domains has been recently identified in *H. longicornis* (Anisuzzaman et al. 2012). The study showed that Longistatin hydrolyzed fibrinogen and activated plasminogen into its active form plasmin (Anisuzzaman et al. 2012). Immunization of Longistatin in animals produced high IgG titers against ticks that revealed 54 % tick repletion, and reduction of tick body weight to 11 % in comparison to controls (Anisuzzaman et al. 2012). Zhou et al. (2006b) cloned and characterized Cystatin gene from *H. longicornis*. Their study revealed that recombinant Cystatin protein showed growth-inhibitory effect on *Babesia bovis* cultured in vitro. The authors indicated that Cystatin is critical for tick immunity (Zhou et al. 2006b).

### Anti-*Dermacentor* Vaccine Candidates

Several species of *Dermacentor* ticks are known carriers of many human pathogens (Table 1). Subolesin, an ortholog of insect akirins is a highly conserved protein that could be potentially considered as vaccine against various blood sucking vectors including ticks and mosquitoes (de la Fuente et al. 2011). Hu et al. (2014) have cloned, characterized and analyzed immunogenicity of *D. silvarum* SUB. Their analysis showed that anti-*D. silvarum* serum recognized recombinant SUB, suggesting an host immune response against this protein (Hu et al. 2014). Fibrinogen-related proteins are shown to be important for tick innate system and are suggested to be potential candidates for the development of anti-tick vaccines (Hajdusek et al. 2013). Recent study has identified fibrinogen-related proteins from *D. marginatus*, *Rhipicephalus appendiculatus*, *R. pulchellus* and *R. sanguineus* (Sterba et al. 2011). Dermacentor ticks feed for 6–10 days with varying expression levels of several tick proteins involved in the process of blood feeding (Sonenshine and Roe 2014). Salivary gland extracts isolated at different times during *D. andersoni* feeding showed esterase activities (Gordon and Allen 1987). Another study determined immunogenic proteins in *R. sanguineus* and *D. reticulatus* ticks using 2-D gel electrophoresis (Vu Hai et al. 2013). IgG response against bite of *R. sanguineus* and *D. reticulatus* ticks were determined in rabbits (Vu Hai et al. 2013). Using antiserum from rabbits exposed to these two ticks, important candidate antigens for the development of anti-tick vaccines were identified (Vu Hai et al. 2013). These include but are not limited to heme-binding, cement and Vitellogenin-2 proteins from *R. sanguineus* and hemelipoglycoprotein precursor, Serpin-2 precursor, Calreticulin from *D. reticulatus* (Vu Hai et al. 2013). The major hemelipoglyco-carrier protein (CP) was identified from *D. variabilis* (Donohue et al. 2008). Expression and domain structure of CP matched Vitellogenin that suggested its role in heme sequestration, evolution of hematophagy and host complementation (Donohue et al. 2008). Anderson et al. (2008) explored *D. variabilis* mialome of ticks and identified 82 transcripts that encode proteins putatively associated in blood meal digestion, enzymes involved in oxidative stress, peptidase inhibitors, protein digestion, protein and lipid binding, mucins and iron/heme metabolism. Transcripts encoding lectin- and allergen-like proteins were also identified in that screen (Anderson et al. 2008). Studies like these would provide evidences on the role of several proteins in blood digestion, antimicrobial activity and transmission of pathogens that could be envisioned for the development of anti-*Dermacentor* vaccines.

### Anti-*Amblyomma* Vaccine Candidates

*Amblyomma* ticks are vectors for several *Rickettsia* spp. and *Francisella tularensis* that cause diseases in humans (Table 1). Mulenga et al. (2013b) have identified tick feeding stimuli responsive gene AamAV422 gene that belong to a novel group of arthropod proteins. AamAV422 contain 14 cysteine amino acid residues that are predicted to form seven disulfide bonds (Mulenga et al. 2013b). AamAV422 has been suggested to mediate tick anti-hemostasis and anti-complement activity (Mulenga et al. 2013b). RNA interference against AamAV422 caused significant (44 %) reduction in tick engorgement weights (Mulenga et al. 2013b). This study concluded that AamAV422 could be considered as a potential candidate for a cocktail or multivalent tick vaccine (Mulenga et al. 2013b). *A. americanum* saliva cysteine proteases have been shown to delay plasma clotting and inhibit platelet aggregation (Mulenga et al. 2013a). Three of the insulin-like growth factor binding proteins (IGFBP) has been identified in *A. americanum* (Mulenga and Khumthong 2010b). Silencing of the IGFBPs had substantial effect on tick feeding (Mulenga and Khumthong 2010b). Organic anion Na^+^-independent transporting polypeptide (AamOatp) that function in wide range of endogenous and xenobiotic compounds transport across plasma membrane has been identified in *A. americanum* (Mulenga et al. 2008). Silencing of AamOatp showed significant reduction in blood intake by ticks (Mulenga et al. 2008). Recently tick CD147 receptor was identified and characterized in *A. americanum* (Mulenga and Khumthong 2010a). CD147 receptors are cell-surface glycoprotein in IgG family that play an important role in extracellular matrix remodeling (Huet et al. 2008). The expression of *A. americanum* CD147 receptor was found to be up regulated upon tick feeding in salivary glands, but remain unaltered in gut suggesting differential roles in these tissues (Mulenga and Khumthong 2010a). The authors of this research study showed that CD147 receptor silencing abrogates tick inter-molt growth (Mulenga and Khumthong 2010a). Study from de la Fuente et al. (2010) have used four cDNA clones encoding putative threonyl-tRNA synthetase (2C9), 60S ribosomal proteins L13a (2D10) and L13e (2B7) and interphase cytoplasm foci protein 45 (2G7) for immunization studies in cattle. Immunization with 2G7 showed significant (*E* > 55 %) control of adult ticks (de la Fuente et al. 2010). Immunization with 2D10, 2G7 and subolesin affected control of both nymphs and adults (de la Fuente et al. 2010).

### Anti-*Rhipicephalus* Vaccine Candidates

*Rhipicephalus* ticks transmit pathogens that cause diseases in humans (Table 1). Study from Rodriguez-Mallon focused on *Rhipicephalus* ribosomal proteins (Rodriguez-Mallon et al. 2012) identified a unique immunogenic region of protein P0. This protein is important in the assembly of 60S ribosomal subunit (Rodriguez-Mallon et al. 2012). The immunogenic region of *Rhipicephalus* species is not conserved in comparison to host P0 protein (Rodriguez-Mallon et al. 2012). Animals were challenged with peptide generated based on the sequence from non-conserved region (Rodriguez-Mallon et al. 2012). The results showed that when larvae were fed on vaccinated animals, significant reduction of viability of newly molted *R. sanguineus* nymphs were noted (Rodriguez-Mallon et al. 2012). More than 90 % reduction was observed in tick egg hatching (Rodriguez-Mallon et al. 2012). Overall, their study provided evidence that vaccination of animals with P0 significantly reduced survival of ticks. *R. microplus* gut cells express Bm86, a membrane-bound glycoprotein on the surface (De La Fuente et al. 2000; Willadsen et al. 1989; Willadsen and Jongejan 1999). Even though the unknown biological function of Bm86 in ticks is yet to be explored, several studies have used Bm86 as a protective antigen against protection from infestation of cattle ticks *R.* *microplus and R. annulatus* (Carreon et al. 2012; de la Fuente et al. 1999; De La Fuente et al. 2000; Rodriguez-Mallon et al. 2012; Rodriguez-Valle et al. 2012; Willadsen and Jongejan 1999; Willadsen et al. 1989, 1996). Protection of Cattle from *R. microplus* infestations by Bm86 vaccination was achieved due to the generation of antigen-specific antibodies by the host that subsequently affected tick infestations and fertility (Willadsen and Jongejan 1999; Willadsen et al. 1989, 1996). Bm86-based TickGARD and Gavac vaccines are now commercially available anti-vector vaccines for animals (Odongo et al. 2007; Valle et al. 2004; Willadsen et al. 1995). Perez-Perez and co-authors vaccinated domestic dogs with Bm86 and infested them with three instars of *R. sanguineus* (Perez-Perez et al. 2010). Their study revealed that ticks fed on vaccinated animals had reduced viability in comparison to controls. *R. sanguineus* was used to evaluate silencing effects of tick protective antigens 4D8 and Rs86, homologues of Bm86 (de la Fuente et al. 2006). Silencing of 4D8 alone had effect on tick feeding, attachment and oviposition (de la Fuente et al. 2006). Silencing of Rs86 had an effect on tick weight and oviposition (de la Fuente et al. 2006). Combined silencing of expression of both genes (4D8 and Rs86) had significant effect on *R. sanguineus* survival, attachment, feeding, weight and oviposition (de la Fuente et al. 2006). The authors from this study proposed the development of multi-antigenic vaccines to prevent infestation from *R. sanguineus* (de la Fuente et al. 2006). This type of multi-antigenic vaccine comprising Vitellin-degrading cysteine endopeptidase and *Boophilus* yolk pro-cathepsin both from *R. microplus* and glutathione S-transferase from *H. longicornis* was evaluated against the cattle tick *R. microplus* (Parizi et al. 2012). Increased antibody titers were evident in vaccinated animals (Parizi et al. 2012). The multi-antigenic vaccine administration in animals had substantial effect on tick infestations, where significantly lower numbers of semi-engorged female ticks were noted (Parizi et al. 2012). Vaccination with truncated construct of cement protein (64TRPs) derived from *R. appendiculatus* showed significant cross protection against *R. sanguineus* (Trimnell et al. 2005). Recently, three major glycoproteins (GLPs) of sizes about 90, 66 and 40 kDa have been purified from adult and larval *Hyalomma dromedarii* (camel ticks) (El Hakim et al. 2011). Immunization with GLPs protected animals from *H. dromedarii* infestations (El Hakim et al. 2011). Immune sera raised against *H. dromedarii* GLPs were able to recognize proteins from *R. sanguineus*, suggesting its efficacy as a potent vaccine candidate against other ticks species (El Hakim et al. 2011). Sialotranscriptome of *R. sanguineus* has now been determined that could aid in the additional discovery of candidate molecules important for the development of anti-*Rhipicephalus* vaccines (Anatriello et al. 2010).

### Anti-*Ornithodoros* Vaccine Candidates

Ornithodoros tick species are important vectors for the transmission of relapsing fever spirochetes (Table 1). Astigarraga et al. (1995) performed a study by immunizing animals with concealed antigens or salivary gland extracts from *O. erraticus*. While immunization with concealed antigens did not result in any protection against ticks, immunization with salivary gland extracts significantly reduced blood ingestion (40–60 %) and fecundity (40–60 %) in ticks (Astigarraga et al. 1995). This was also evident in immunization with salivary gland extracts prepared from *O. moubata* (Astigarraga et al. 1995). Factor Xa inhibitor (fXaI; anti-coagulation factor) has been identified in *O. savignyi* that shows 46 % identity and 78 % similarity in amino acid sequence with fXaI from *O. moubata* (Joubert et al. 1998). Midgut membrane extracts prepared from *O. erraticus* were administered to mice and pigs to test protective responses in these animals against ticks (Manzano-Roman et al. 2006). When midgut extracts were injected with Freund’s adjuvants into these animals, 80 % of the immature forms of the ticks were killed in the first 72 h post-feeding and also decreased fecundity in females by more than 50 % (Manzano-Roman et al. 2006). The significant rate of killing was attributed to the damage of midgut wall of ticks (Manzano-Roman et al. 2006). It was also noted that some proteins present in the midgut extracts were expressed in all developmental stages of *O. erraticus* ticks and found to be induced upon feeding suggesting important targets for the vaccine development (Manzano-Roman et al. 2006). Subolesin orthologs were identified in *O. erraticus* and *O. moubata* ticks (Manzano-Roman et al. 2012b). The Subolesin of both these ticks show more than 69 and 74 % identify with Subolesin from hard ticks in their nucleotide and amino acid sequences, respectively (Manzano-Roman et al. 2012b). RNAi-mediated silencing of Subolesin inhibited *Ornithodoros* oviposition indicating importance of this protein in tick oviposition (Manzano-Roman et al. 2012b). Immunization studies with soft tick recombinant Subolesin induced partial protective effect resulting in reduced oviposition rates of these ticks (Manzano-Roman et al. 2012b). A lectin, designated as Dorin M, with high hemagglutinating activity was isolated from *O. moubata* (Kovar et al. 2000). This was a first report of purified lectin from ticks (Kovar et al. 2000). Significant levels of enolase have been found in the saliva of *O. moubata* ticks (Diaz-Martin et al. 2013). Enolase is a glycolytic enzyme that acts as plasminogen receptor on cell surface to promote fibrinolysis and extracellular matrix degradation (Diaz-Martin et al. 2013). The function of enolase could be important for ticks to digest clots and also to prevent clotting formed during feeding (Diaz-Martin et al. 2013). RNAi and immunization studies revealed that *O. moubata* enolase could be involved in tick reproduction (Diaz-Martin et al. 2013). Using self-assembled protein arrays, a recent study has identified important tick molecules in the *O. moubata* salivary gland expression library (Manzano-Roman et al. 2012a). Several clones that bind to human P-selectin were identified in the screen (Manzano-Roman et al. 2012a). Secreted tick phospholipase A2, which could function as potential new ligand for P-selectin was also identified (Manzano-Roman et al. 2012a). This strategy provides one of the important breakthroughs in the identification of several vaccine candidates in ticks (Manzano-Roman et al. 2012a). Cystatin from *O. moubata* has been identified (Salat et al. 2010). Immunization studies with recombinant Cystatin (OmC2) significantly suppressed survival of *O. moubata* (Salat et al. 2010). A tick salivary gland glycoprotein of 44 kDa (Om44) that carries heparin determinant has been identified in *O. moubata* salivary gland extracts (Garcia-Varas et al. 2010). Partial characterization of this protein revealed that Om44 binds host P-selectin, that presumably prevents the adhesion of leucocytes and platelets to vessel walls (Garcia-Varas et al. 2010). Om44 cannot be recognized by host immune system, but can be neutralized by antibodies. This study found significant inhibition of *O. moubata* feeding on vaccinated animals in comparison to the controls, making new prospects in the development of anti-*O. moubata* and perhaps other *Ornithodoros* species vaccine (Garcia-Varas et al. 2010).

## Conclusions

In this review, we provide an overview of potential candidates for the development of anti-vector vaccines against human diseases transmitted by various hard and soft ticks. Several commercial and technical challenges need to be overcome in order to develop an effective anti-vector vaccine for human use (de la Fuente et al. 2007; Merino et al. 2013). Some of the challenges to overcome are: (1) limitation in the molecular evidences to understand how anti-vector vaccine works in humans; (2) overall ineffectiveness of anti-vector vaccines to control certain tick population (De La Fuente et al. 2000; de Vos et al. 2001); (3) limited interest for commercialization and vaccine production on large scale and (4) insufficient knowledge dispersal to the public about the use of anti-vector vaccines.

Over the past decade, several studies have used genomics, transcriptomics and proteomics approaches to identify some of the potential candidates for the development of anti-vector vaccines (Dinglasan et al. 2005; Geiger et al. 2011; Gibson et al. 2013; Heekin et al. 2013a, b; Hill et al. 2005; Patramool et al. 2012; Ramabu et al. 2010; Schwarz et al. 2013; Valenzuela 2004b; Vernick and Waters 2004; Villar et al. 2014a, b). With the availability of already abundant amount of information on several tick genomes, transcriptomes and proteomes, future studies need to focus on developing suitable animal models and approaches to evaluate proper efficacy of a molecule as a vaccine candidate for human trials. Studies using multidisciplinary approaches such as use of bioinformatic analysis for epitope prediction, protein folding and post-translational modifications on a vaccine candidate molecule would strengthen development of anti-vector vaccines. In addition, development of multivalent-vaccine formulations comprising vaccines targeting both vector and pathogen would provide a significant leap in this research area to move forward from bench-to-bedside.
